# Longitudinal micro-computed tomography-derived biomarkers quantify non-resolving lung fibrosis in a silicosis mouse model

**DOI:** 10.1038/s41598-020-73056-6

**Published:** 2020-09-30

**Authors:** Kaat Dekoster, Tatjana Decaesteker, Nathalie Berghen, Sofie Van den Broucke, Anne-Charlotte Jonckheere, Jens Wouters, Anton Krouglov, Rik Lories, Ellen De Langhe, Peter Hoet, Erik Verbeken, Jeroen Vanoirbeek, Greetje Vande Velde

**Affiliations:** 1 Department of Imaging and Pathology, Biomedical MRI/MoSAIC, KU Leuven, Leuven, Belgium; 2 Department of Chronic Diseases, Metabolism and Ageing, Lab of Respiratory Diseases, KU Leuven, Leuven, Belgium; 3 Department of Development and Regeneration, Skeletal Biology and Engineering Research Center, KU Leuven, Leuven, Belgium; 4 Division of Rheumatology, University Hospitals Leuven, Leuven, Belgium; 5 Department of Public Health and Primary Care, Centre for Environment and Health, KU Leuven, Leuven, Belgium; 6 Department of Microbiology, Immunology and Transplantation, Allergy and Clinical Immunology Research Group, KU Leuven, Leuven, Belgium; 7 Department of Imaging and Pathology, Translational Cell and Tissue Research Unit, KU Leuven, Leuven, Belgium

**Keywords:** Physiology, Biomarkers, Diseases, Medical research

## Abstract

In spite of many compounds identified as antifibrotic in preclinical studies, pulmonary fibrosis remains a life-threatening condition for which highly effective treatment is still lacking. Towards improving the success-rate of bench-to-bedside translation, we investigated in vivo µCT-derived biomarkers to repeatedly quantify experimental silica-induced pulmonary fibrosis and assessed clinically relevant readouts up to several months after silicosis induction. Mice were oropharyngeally instilled with crystalline silica or saline and longitudinally monitored with respiratory-gated-high-resolution µCT to evaluate disease onset and progress using scan-derived biomarkers. At weeks 1, 5, 9 and 15, we assessed lung function, inflammation and fibrosis in subsets of mice in a cross-sectional manner. Silica-instillation increased the non-aerated lung volume, corresponding to onset and progression of inflammatory and fibrotic processes not resolving with time. Moreover, total lung volume progressively increased with silicosis. The volume of healthy, aerated lung first dropped then increased, corresponding to an acute inflammatory response followed by recovery into lower elevated aerated lung volume. Imaging results were confirmed by a significantly decreased Tiffeneau index, increased neutrophilic inflammation, increased IL-13, MCP-1, MIP-2 and TNF-α concentration in bronchoalveolar lavage fluid, increased collagen content and fibrotic nodules. µCT-derived biomarkers enable longitudinal evaluation of early onset inflammation and non-resolving pulmonary fibrosis as well as lung volumes in a sensitive and non-invasive manner. This approach and model of non-resolving lung fibrosis provides quantitative assessment of disease progression and stabilization over weeks and months, essential towards evaluation of fibrotic disease burden and antifibrotic therapy evaluation in preclinical studies.

## Introduction

Interstitial lung diseases (ILDs) are a heterogeneous group of lung diseases characterized by the presence of cellular proliferation, cellular infiltration and/or fibrosis of the lung parenchyma not due to infection or neoplasia^1,2^. Depending on the cause of disease, different types of ILD can be identified: e.g. hypersensitivity pneumonitis, pulmonary sarcoidosis, silicosis or ILD secondary to an underlying autoimmune disease most of which remain untreatable^1^.

Although multiple therapeutic compounds have shown promising results in preclinical models of pulmonary fibrosis, only few of them have been successfully translated to the clinic^3–5^. Several challenges in the research field may explain this translational problem. Animal models are indispensable to fibrosis and therapy research but have the inherent limitation that no model exactly mimics the human disease^6,7^. Second, in spite of technological advancements^8–10^, only endpoint measurements (biochemical assessment of collagen content, gene expression, histology and lung function) are typically used to evaluate the burden of lung disease and drug efficacy. These often come with high variability, only provide a snapshot of a certain stage of the disease and are unable to give temporal and spatial information of the pathology over the entire lung. Moreover, they differ substantially from the endpoints used in clinical studies [e.g. computed tomography, lung functional readouts such as forced vital capacity (FVC), forced expiratory volume (FEV_1_) and Tiffeneau index (FEV_1_/FVC)], stressing the need for more clinically relevant readouts which would improve comparing pathology in human ILD and animal models thereof, and the preclinical screening efficiency of new therapeutics. Lastly, most often only prophylactic testing of the drug is performed because the currently most used mouse models and readouts only allow for this type of set-up due to their limited and/or variable fibrotic phase. Therapeutic testing when fibrosis is appearing or omnipresent would better mimic the real-life situation and be more relevant to evaluate the antifibrotic properties of a putative compound^3^.

To evaluate interventions in a truly therapeutic regime, we need to know if, when and how much fibrosis and inflammation are present at the time of drug administration. This is only possible by the use of non-invasive techniques such as imaging by micro-computed tomography (µCT), in combination with a not spontaneously resolving fibrotic disease model. The often-used acute bleomycin model is characterized by spontaneous regression of fibrosis, an observation that does not reflect reality in human patients. Chronic models of lung fibrosis such as the silica-induced pulmonary fibrosis model better resemble this persistent aspect of human disease and may therefore be a better model for preclinical studies.

µCT has already proven to have important benefits for longitudinal evaluation of disease in preclinical animal models^8,9,11–14^. Due to its high air-tissue contrast, it is a particularly excellent technique to study and quantify lung disease burden such as pulmonary fibrosis^8,14–17^, whereby its added non-invasive value to standard endpoint readouts such as histology and hydroxyproline assays has been established. Yet, most often µCT is still not implemented in preclinical therapy studies in spite of its capacity to longitudinally visualize and quantify the inflammatory and fibrotic process for each animal individually, thereby identifying the window for therapeutic intervention.

Bridging the gap between preclinical and clinical research by assessing quantitative and clinically relevant CT- and lung function-derived readouts in a relevant animal model would have critical impact on research efficiency in identifying therapeutic targets and compounds, ultimately leading to better patient care. Therefore, we here (1) evaluate the silica-induced pulmonary fibrosis model with in vivo and ex vivo readouts and postulate it to be a model more relevant to human disease; (2) we assess the added value of longitudinal imaging using µCT to provide non-invasive biomarkers characterizing pulmonary fibrosis progression in mouse and (3) we correlate in vivo µCT-derived biomarkers with endpoint measurements of lung function, host response and lung fibrosis, thereby addressing analogies and dissimilarities between mouse and human fibrotic lung disease regarding interpretation of clinically relevant readouts.

## Methods

### Animal model

All animal experiments were carried out in compliance with national and European regulations and were approved by the animal ethics committee of KU Leuven (P057/2018). Mice were kept in a conventional animal facility with individually ventilated cages and free access to food and water. Eight-week-old male C57Bl/6JRj mice (Janvier, Le Genest, France) were oropharyngeally instilled with crystalline silica particles (5 mg/animal), kindly provided by B Fubini (Facoltà di Farmacia, Università di Torino, Italy), or saline (NaCl 0.9%). Silica particles were baked for 2 h at 180 °C and dissolved in saline with a concentration of 0.125 mg/µL. Next, the solution was probe sonicated for 16 min. Animals were anesthetized by inhalation of 5% isoflurane (Piramal Healthycare, Morpeth, Northumberlang, United Kingdom) in 100% oxygen. By simultaneously holding the tongue and covering the nose, animals were oropharyngeal instilled with 40 µL of silica/saline^18,19^.

### Experimental design

**Figure 1:**
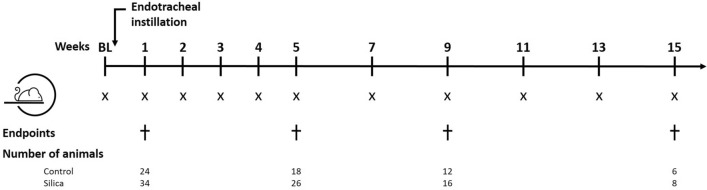
Experimental design. This scheme summarizes the followed protocol. X indicates a µCT scan. At four endpoints (1, 5, 9 and 15 weeks post instillation), a cohort of animals was sacrificed to obtain lung function and ex vivo measurements. At each endpoint, the number of animals is indicated and represents the number of animals up until that endpoint.

Mice were randomly divided over treatment groups. Animals were scanned at baseline, weekly for the first 5 weeks and every 2 weeks for the following 10 weeks. At 1, 5, 9 and 15 weeks after silica instillation, a randomly selected cohort of the animals was sacrificed for endpoint measurements (Fig. 1).

### Micro-computed tomography

Mice were anesthetized by inhalation of 1.5–2% isoflurane in 100% oxygen and scanned in supine position using a dedicated in vivo μCT scanner (Skyscan 1278, Bruker µCT, Kontich, Belgium). The following parameters were used: 50 kVp X-ray source voltage and 918 μA current combined with a composite X-ray filter of 1 mm aluminium, 55 ms exposure time per projection, acquiring projections with 0.9° increments over a total angle of 220°, and 10 cm field of view covering the whole body producing reconstructed 3D data sets with 50 μm isotropic reconstructed voxel size. The data was acquired with retrospective respiratory gating; images were acquired in list mode with 9 projections per view, retrospectively time-based sorted, resulting in four reconstructed 3D data sets corresponding to four different phases of the breathing cycle (4D). We here report data at the end of the expiratory phase.

Software provided by the manufacturer (TSort, NRecon, DataViewer, and CTan) was used to retrospectively gate, reconstruct, visualize, and process μCT data as described previously^20,21^. For Hounsfield unit (HU) calibration, a phantom was scanned consisting of an air-filled 1.5 mL tube inside a water-filled 50 mL tube. Based on full stack histograms of a volume-of-interest (VOI) containing only water or air, the main grayscale index of water (133) was set at 0 HU and grayscale index of air (9) at − 1000 HU. Quantification of the mean lung density, non-aerated lung volume, aerated lung volume, and total lung volume was carried out for a VOI covering the lung, manually delineated on the coronal μCT images, avoiding the heart and main blood vessels. When defining the VOI covering the lungs, we define the top of the lungs by the CT section defined by the point where the clavicle touches the sternum and the bottom by a plane under the lungs through the liver. The threshold used to distinguish aerated from non-aerated lung tissue volume was manually set at − 223 HU and kept constant for all data sets.

### Pulmonary function

Lung function was assessed at 1, 5, 9 and 15 weeks after silica instillation using the flexiVent FX system (SCIREQ, EMKA Technologies, Montreal, Canada). The system was designed to concurrently measure forced oscillations and forced expiration parameters, as described previously^10^. Briefly, the system operated with flexiWare 7.6 software and was equipped with a FX1 module, a negative pressure forced expiration (NPFE) extension for mice. Mice were anesthetized with an intraperitoneal injection of pentobarbital (70 mg/kg BW, Nembutal). Once sufficiently anesthetized, a tracheotomy was performed to insert an 18-gauge metal cannula. Mice were quasi-sinusoidal ventilated with a tidal volume of 10 mL/kg and a frequency of 150 breaths/min to mimic spontaneous breathing.

At the start of the experiment, two successive deep inflations were applied to maximally inflate the lungs to a pressure of 30 cm H_2_O in order to open the lungs and determine the inspiratory capacity (IC). Between each set of perturbation, a deep inflation was applied. Next, the forced oscillation perturbation ‘Prime-8’ (P8) that generates forced oscillations with a frequency between 1 and 20.5 Hz during 8 s, was executed five times. The reported central airway resistance (Rn), tissue damping (G), tissue elasticity (H) and tissue hysteresivity (eta = G/H) are the average of three measurements. Next, a NPFE (negative pressure forced expiratory flow) perturbation was performed to measure the peak expiratory flow (PEF), forced vital capacity (FVC) and forced expiratory volume in 0.1 s (FEV_0.1_). Reported values are the average of three accepted measurements (COD > 0.9) for each individual data point.

### Inflammatory and permeability evaluations

Immediately after the lung function measurements, plasma was collected to measure surfactant protein (SP)-D concentration by ELISA (Duoset ELISA, R&D Systems Inc., DY6839-05) according to the manufacturers’ instructions. The whole lung was lavaged three times in situ with 0.7 mL sterile saline (0.9% NaCl), the collected fluid pooled and analysed for total and differential cell counts. The total number of cells in the BAL fluid was counted using a Bürker hemocytometer. Subsequently the bronchoalveolar lavage (BAL) fluid was centrifuged (1000 g, 4 °C, 10 min) and the supernatant collected and frozen (− 80 °C) until further analysis. For differential cell counts, the cells were resuspended in 1 mL saline (0.9% NaCl), of which 250 µL of the resuspended cells were spun (300 g, 4 °C, 6 min; Cytospin 3, Shandon, TechGen, Zellik, Belgium) onto microscope slices, air-dried and stained (Diff-Quik method, Medical Diagnostics, Düdingen, Germany). For each sample, 200 cells were counted to quantify the number of macrophages, eosinophils, neutrophils and monocytes. Protein concentration was determined on the cell-free BAL supernatant using the Bradford method with bovine serum albumin as a standard. Cytokine counts (IFN-γ, IL-10, IL-13, IL-1β, IL-17A, IL-17F, IL-6, MCP-1, MIP-2, TNF-α, TGF-β1, TGF-β2 and TGF-β3) were determined on the cell-free BAL supernatant using a U-PLEX Biomarker Assay and U-PLEX TGF-β Combo Assay (Meso Scale Discovery, Maryland, USA) according to manufacturers’ instructions.

### Histopathological and histochemical analysis

The left lung lobe was manually inflated up until physiological TLC was reached, fixed with 4% paraformaldehyde and paraffin embedded. 5 µm sagittal sections were stained with haematoxylin–eosin (H&E) and Sirius red and examined with polarized light to visualize the silica particles and collagen formation.

The right lung lobes were clamped, collected and stored at − 80 °C for hydroxyproline quantification as previously described^22^. In short, right lung lobes were hydrolysed for 3 h in 6 M HCl at 120 °C. After cooling down for 15 min, the pH was neutralized (pH 6–7) using 500 ml NaOH. Free hydroxyproline was oxidized with Chloramine-T for 20 min after which the oxidation reaction was stopped using 70% perchloric acid. Ehrlich’s reagent was added, and samples were heated for 20 min in a 60 °C water bath. After cooling down, absorbance was measured at 570 nm and concentrations were calculated based on a standard curve.

### Statistical analyses

The data were analysed using GraphPad Prism (version 8.02. Graphpad Software Inc., San Diego, USA). The data are presented as mean with standard deviation (SD). Normal distribution was assessed by the Kolmogorov–Smirnov test and visual inspection of the QQ-plots. For cross-sectional data with a balanced design, two-way ANOVA with Sidak’s multiple comparison post hoc test was performed. Two-way ANOVA with Sidak’s correction for multiple comparison was based on the following number of animals: w1 6 control and 6 silica mice; w5 6 control and 10 silica mice; w9 6 control and 8 silica mice; w15 6 control and 8 silica mice. Data of longitudinal µCT-derived biomarkers (with missing values and an unbalanced design; mean lung density, total lung volume, non-aerated lung volume and aerated lung volume) were analysed using a mixed-effects model with Geisser–Greenhouse correction, for assessing the interaction between time and treatment. For multiple comparison, Sidak’s post hoc test was used. Mixed-effects model with Geisser–Greenhouse correction was based on the following number of animals for every timepoint: BL-w1 24 control and 34 silica mice; w2-5 18 control and 26 silica mice; w7-9 12 control and 16 silica mice; w11-15 6 control and 8 silica mice. For correlation analysis, the Pearson correlation coefficient was determined. For all tests, statistical significance was assumed when *p value < 0.05; **p value < 0.01 and ***p value < 0.001.

## Results

### In vivo µCT non-invasively captures the non-resolving inflammatory and fibrotic response to silica instillation

Mice were instilled with silica or vehicle, scanned at baseline and longitudinally for a period of 15 weeks with respiratory-gated high-resolution low-dose µCT. Silica-instilled mice initially lost weight, then fully recovered and regained normal body weight within 7–14 days without signs of distress (Fig. S1). Survival rate was 100%.

**Figure 2:**
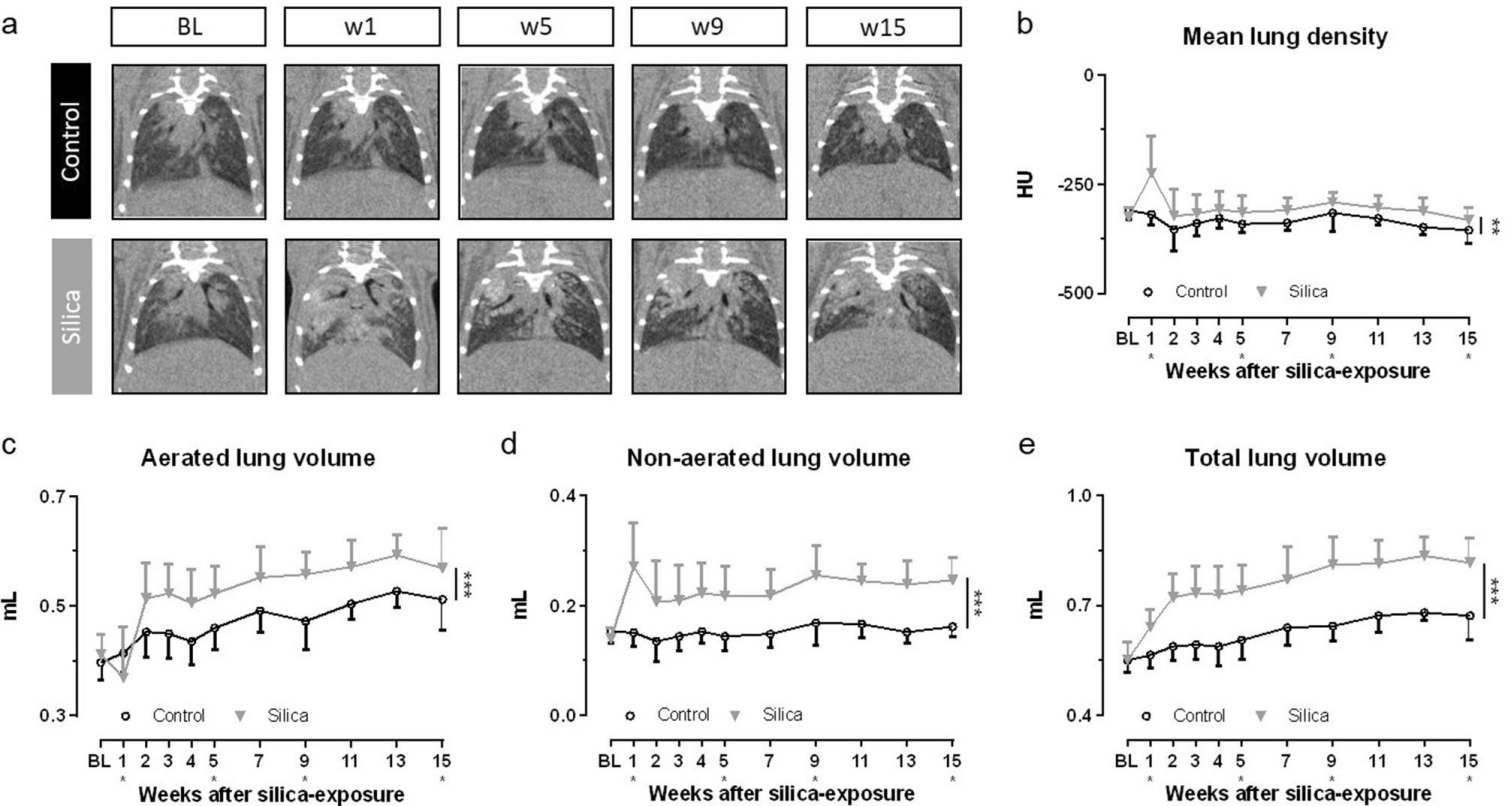
µCT visualizes and quantifies onset and progression of the inflammatory and fibrotic response upon silica-instillation. (**a**) Representative images of one control and one silica-instilled animal at selected time points, from baseline up until 15 weeks, visualize the inflammatory and fibrotic response. µCT-derived biomarkers reflect disease onset and progression in silica-instilled animals: (**b**) mean lung density expressed in HU (**c**) aerated lung volume (mL) (**d**) non-aerated lung volume (mL) (**e**) total lung volume (mL). Data are presented as mean ± SD. Full line represents longitudinal data. Mixed-effects model with Geisser–Greenhouse correction was based on the following number of animals for every timepoint: BL-w1 24 control and 34 silica mice; w2–5 18 control and 26 silica mice; w7–9 12 control and 16 silica mice; w11–15 6 control and 8 silica mice. *p value < 0.05; ***p value < 0.001; *BL* baseline; *under the x-axes indicate endpoints.

µCT showed large hyperdense areas that were most pronounced 1 week after silica-instillation, most likely corresponding to acute inflammatory processes (Fig. 2a). Past the first week, the presence of hyperdense areas remained extensive but their volume and density decreased to then remain stable, coinciding with the regain of body weight. Quantification of longitudinal µCT-derived biomarkers corroborated these visual observations (Fig. 2b–e). Silica instillation resulted in a significantly increased mean lung density starting at week 1 that remained present until endpoint. The non-aerated lung volume, which directly quantifies inflammatory and fibrotic disease burden^21^, also peaked at week 1 and remained significantly and stably increased up until endpoint. The aerated lung volume transiently decreased at week 1 to steadily increase at elevated levels thereafter, in contrast to loss of aerated lung volume previously observed in the bleomycin model^8^. The increase in aerated and non-aerated lung volume is paralleled by the total lung volume that is progressively increased up until endpoint in silica-instilled mice, in contrast to control mice where small lung volume changes only reflect normal changes with aging^21^.

### Silica instillation significantly alters lung function

**Figure 3:**
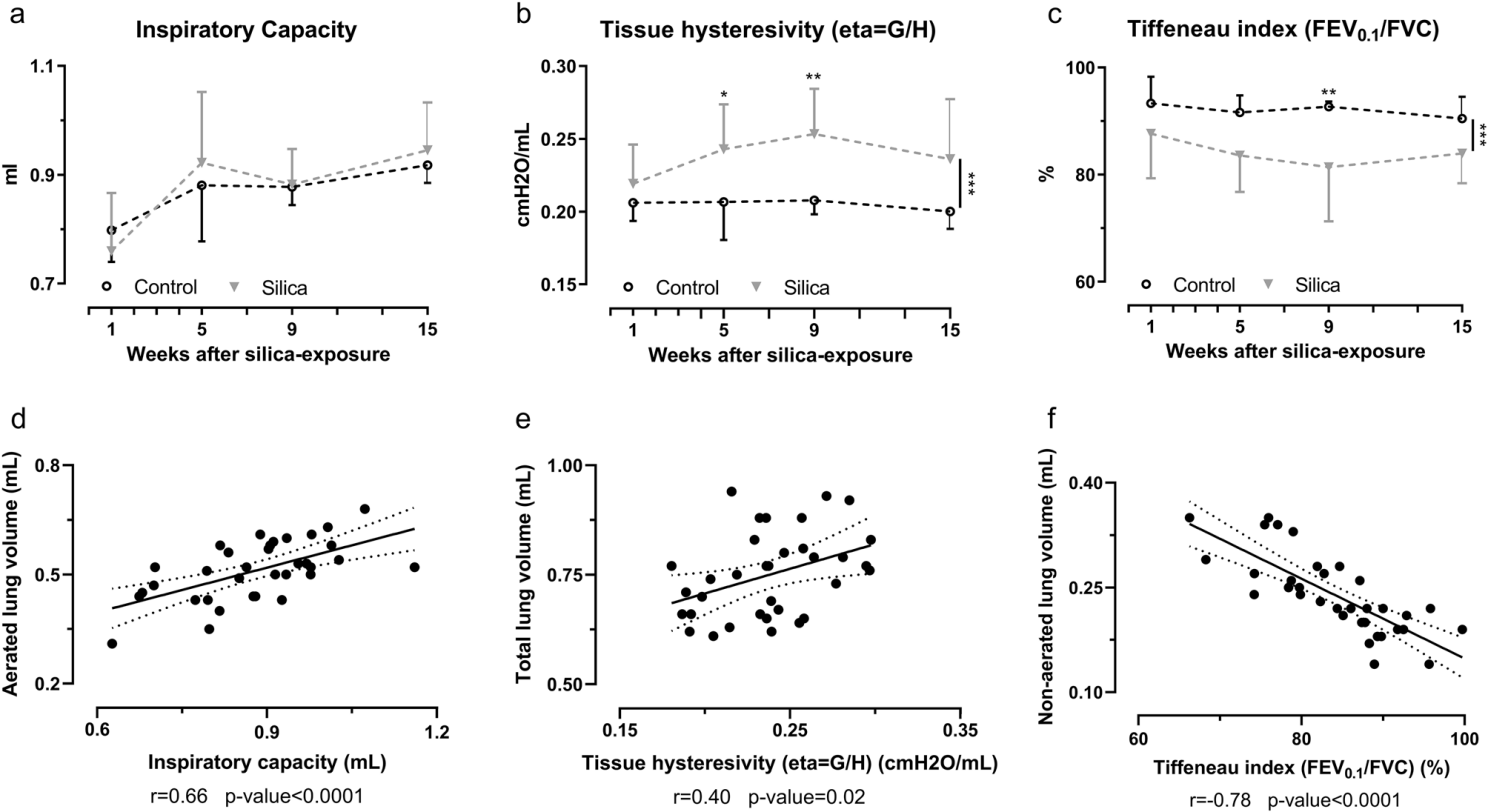
Pulmonary function data reflect fibrotic disease and correlate with in vivo µCT-derived biomarkers. At every endpoint, lung function was assessed by performing (**a**) deep inflation, (**b**) forced oscillations and (**c**) negative pressure-driven forced expiration maneuvers. Data are presented as mean ± SD. Dashed line represents cross-sectional data. Two-way ANOVA with Sidak’s correction for multiple comparison was based on the following number of animals: w1 6 control and 6 silica mice; w5 6 control and 10 silica mice; w9 6 control and 8 silica mice; w15 6 control and 8 silica mice. *p value < 0.05; **p value < 0.01; ***p value < 0.001. Correlations between µCT-derived biomarkers and lung function measurements indicate the agreement between both methods (**d**) aerated lung volume and inspiratory capacity (**e**) total lung volume and tissue hysteresivity (**f**) non-aerated lung volume and Tiffeneau index. For every correlation the Pearson correlation coefficient and the *p* value are given. Correlations only include silica-instilled animals.

We cross-validated our in vivo µCT findings with lung function readouts at endpoint from sub-cohorts of animals at week 1, 5, 9 and 15 after instillation. These timepoints were chosen to cover the initial inflammatory phase (week 1) followed by the fibrotic phase (week 5–15). The inspiratory capacity of silica mice was unaltered compared to controls (Fig. 3a) and correlated with the aerated lung volume derived from the µCT scans (r = 0.6612, *p* value < 0.0001) (Fig. 3d). The airway resistance, tissue damping and tissue elasticity were not different after silica-instillation (Fig. S3a–f), but the tissue hysteresivity, reflecting tissue inhomogeneity’s and structural changes in the lungs, was significantly increased and correlated with the total lung volume (r = 0.4027, *p* value = 0.0182) (Fig. 3b–e). The Tiffeneau index (FEV_0.1_/FVC) was significantly decreased after silica instillation and correlated with the non-aerated lung volume (r = 0.7764, *p* value < 0.0001) (Fig. 3c–f). Contrary to what would be expected in human restrictive disease but consistent with the µCT-derived lung volumes for this model, peak expiratory flow, forced vital capacity and forced expiratory flow in 0.1 s were not affected in this mouse model (Figure S3g–l).

### Ex vivo readouts confirm the functional and imaging-based detection of inflammatory and fibrotic responses

**Figure 4:**
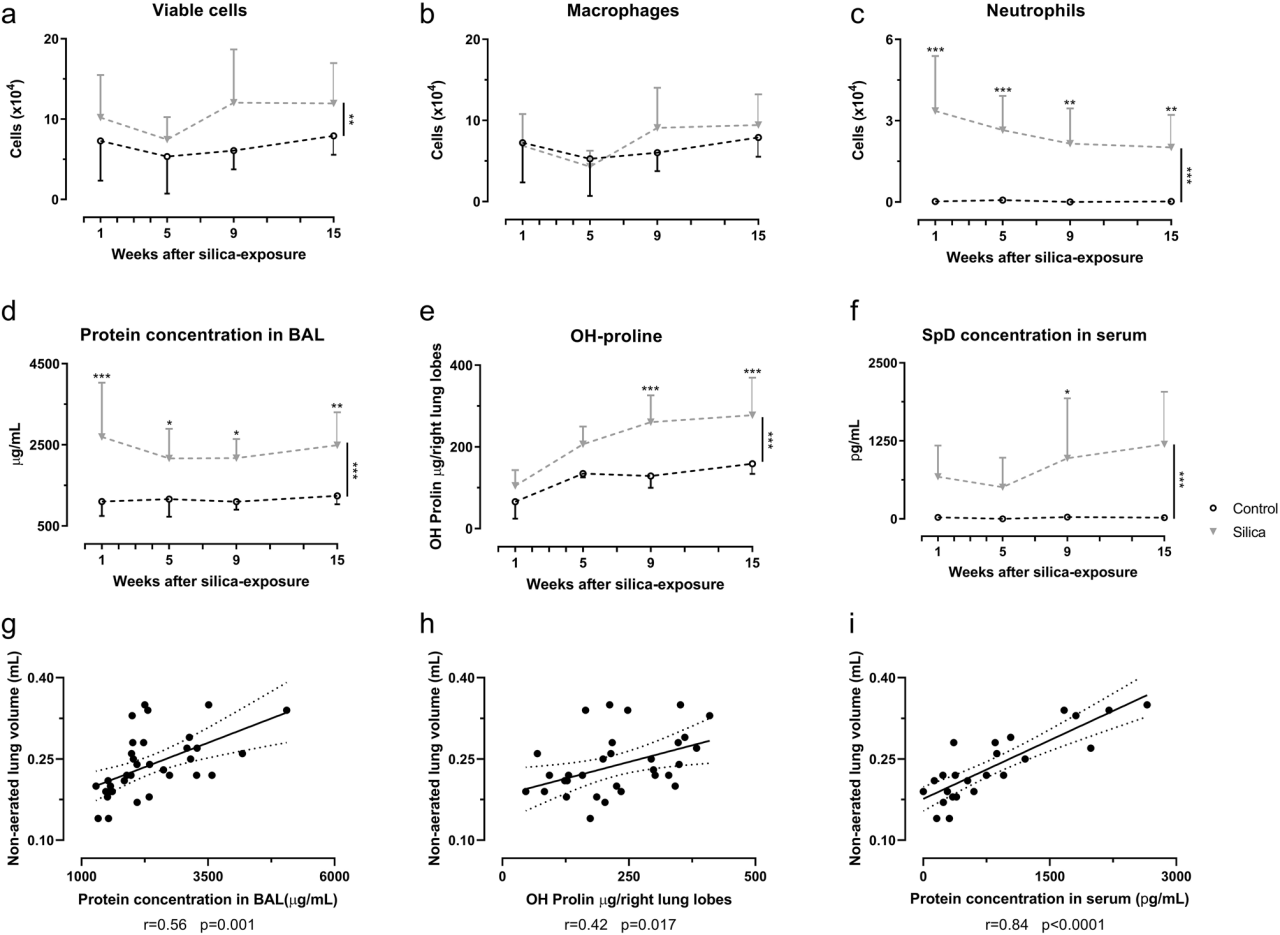
Silica-instillation changes inflammatory readouts of BAL fluid and serum and increases the collagen content. At every endpoint, several ex vivo readouts for inflammatory and fibrotic responses were obtained. Silica instillation changed the number of viable cells (**a**) and neutrophils (**c**) but did not alter the number of macrophages (**b**) in the BAL fluid. Moreover, silica increased the protein concentration in the BAL fluid (**d**), the collagen content measured by OH-proline assay reflecting a fibrotic response on the silica particles (**e**) and the surfactant protein D (SpD) concentration in serum (**f**). Data are presented as mean ± SD. Dashed line represents cross-sectional data. Two-way ANOVA with Sidak’s correction for multiple comparison was based on the following number of animals: w1 6 control and 6 silica mice; w5 6 control and 10 silica mice; w9 6 control and 8 silica mice; w15 6 control and 8 silica mice. *p value < 0.05; **p value < 0.01; ***p value < 0.001. Correlations between µCT-derived biomarkers and ex vivo measurements indicate the agreement between the inflammatory and fibrotic disease and µCT-derived biomarkers (**g**) non-aerated lung volume and protein concentration in bronchoalveolar lavage fluid, (**h**) non-aerated lung volume and OH-proline content, (**i**) non-aerated lung volume and protein concentration in serum. For every correlation the Pearson correlation coefficient is given and the *p* value. Correlations only include silica-instilled animals.

Cellular analysis of the BALF showed a significant neutrophilic inflammation that remained elevated up until 15 weeks (20% neutrophils of total cells at 15 weeks, Fig. S2) and did not differ significantly between time points (Fig. 4a–c, Fig. S2). The number of macrophages was unaffected. Additionally, protein concentration in BALF increased (Fig. 4d), indicative of the inside-out leaky epithelium. Surfactant protein D (SpD) concentration in serum was significantly elevated upon silica instillation, which points to outside-in leakage (Fig. 4f). The hydroxyproline content, reflecting the amount of collagen in lung tissue, was significantly elevated from 9 weeks on (Fig. 4e). Both the higher inflammatory and fibrotic burden determined from the µCT scans (non-aerated lung volume) correlated with a higher protein concentration in BALF (r = 0.5557, *p* value = 0.0006), serum (r = 0.8445, *p* value < 0.0001) and OH-proline content (r = 0.4187, *p* value = 0.0171) (Fig. 4g–i).

**Figure 5:**
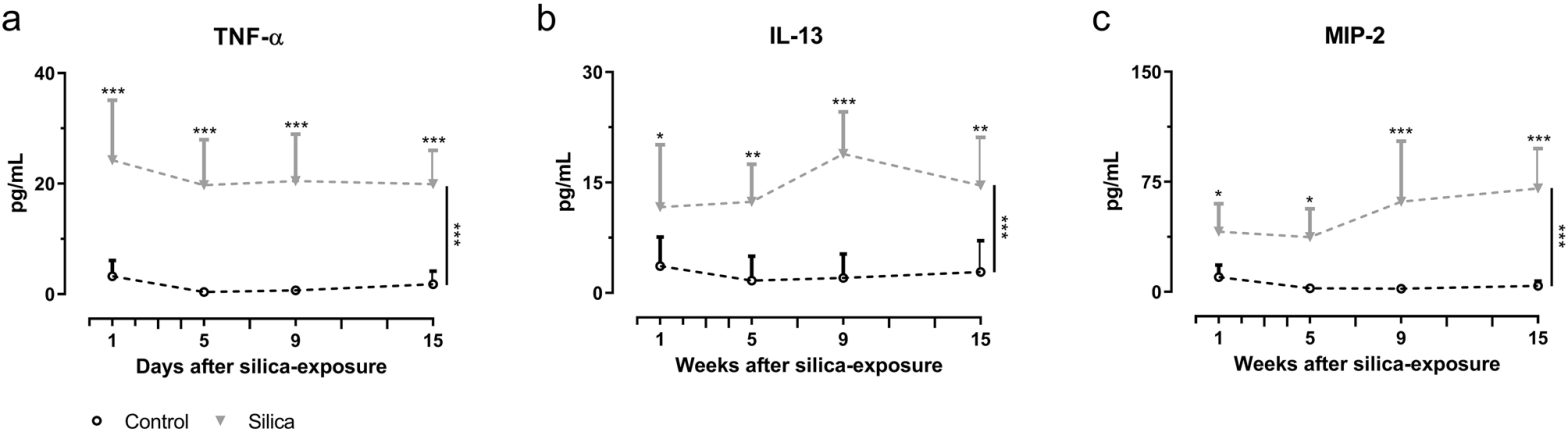
Silica instillation induces a pro-inflammatory response characterized by an increased TNF-α, IL-13 and MIP-2 content in BAL fluid. At every endpoint, cytokine concentration was determined. Silica-instillation altered the amount of (**a**) TNF-α, (**b**) IL-13 and (**c**) MIP-2 concentration in the BAL fluid. Data is presented as mean ± SD. Dashed line represents cross-sectional data. Two-way ANOVA with Sidak’s correction for multiple comparison was based on the following number of animals: w1 6 control and 6 silica mice; w5 6 control and 10 silica mice; w9 6 control and 8 silica mice; w15 6 control and 8 silica mice. *p value < 0.05; **p value < 0.01; ***p value < 0.001.

Further characterizing the time course of the inflammatory and fibrotic response to silica-induction revealed that silica instillation resulted in a significant elevation of type 2 cytokine IL-13 and pro-inflammatory cytokines TNF-α and MIP-2 levels (Fig. 5a–c). Moreover, we observed an overall increase of type 1 cytokine IFN-γ, type 2 cytokine IL-10, type 3 cytokines IL-17A and IL-17F, pro-inflammatory cytokines IL-1β and IL-6, chemokine MCP-1 and pro-fibrotic cytokines TGF-β1 and TGF-β2 (Fig. S4a-i). No significant difference was found for TGF-β3 (Fig. S4j).

**Figure 6:**
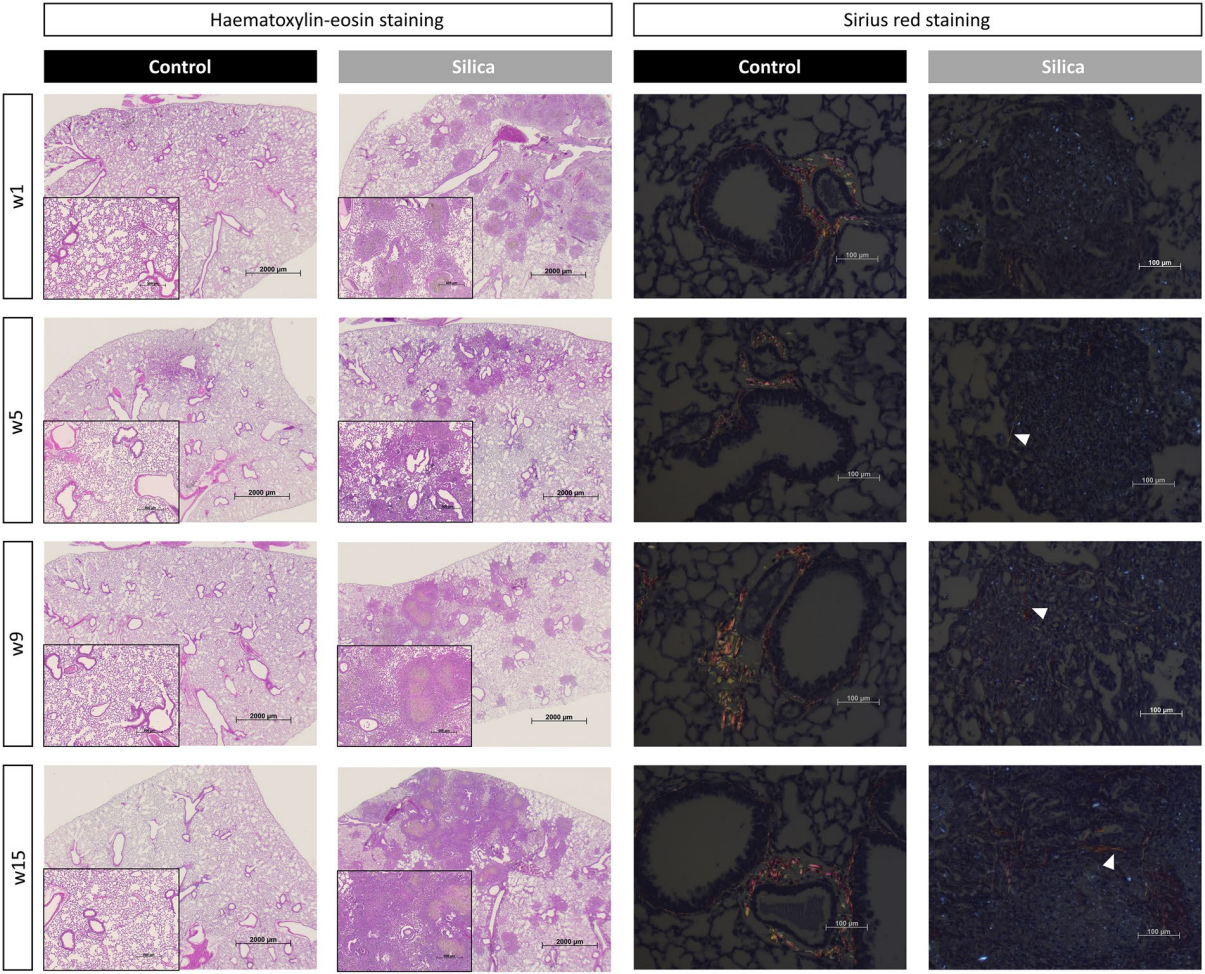
Histopathological analysis of silica-instilled animals at every timepoint. Representative H&E stained images are shown for every timepoint for control and silica-instilled animals (magnification of 12.5 × and 50 ×). Representative Sirius Red images are shown for every timepoint for control and silica-instilled animals (magnification of 200 ×). For control animals, only native collagen is shown in centrilobular area. For silica-instilled animals, centrilobular located nodules with increasing amount of newly formed collagen (indicated with white arrows) are shown.

One week after silica-instillation, histopathological examination showed a general, mostly centrilobular located inflammatory reaction that was completely absent in controls (Fig. 6 and Fig. S5A–G). Silica particles were encapsulated with neutrophils, macrophages, proteins and wound fluid. Only native collagen was observed at this timepoint, same as in control animals. Five weeks after silica instillation, polarization microscopy visualized newly formed collagen indicating a fibrotic pattern consisting of several fibrotic nodules, located centrilobularly. The same type of pathology was observed at 9- and 15-weeks post instillation indicating long-lasting fibrosis, whereas controls did not show any inflammatory response nor fibrotic nodules at any time point.

## Discussion

More and more attention has been given to the disease silicosis lately, as it affects millions of people in India, China, Australia, Turkey and USA, emphasizing the importance of silica-induced pulmonary fibrosis research^23–25^. Clinically relevant readouts delivered by translational techniques such as µCT and lung function measurements would add to the current standard methodology used to evaluate disease process and therapeutic effects in pulmonary fibrosis mouse models^3^. In this study, we show that in vivo longitudinal µCT-derived biomarkers identify and quantify lung inflammatory and fibrotic disease in the context of silicosis, its onset and progress, which is paramount over endpoint readouts and will allow precise timing of possible future antifibrotic therapy testing.

We selected a silica-induced pulmonary fibrosis model for which we identified a steady-state fibrotic phase of 10 weeks in which interventions can be tested. We cross-validated with in vivo µCT-derived biomarkers, lung function and routinely used ex vivo read-outs that fibrosis did not spontaneously resolve. This study is the first to demonstrate the power of µCT to deliver non-invasive biomarkers that quantify in a mouse model of silica-induced fibrosis in a longitudinal manner the degree of inflammation and fibrosis, on top of providing visual information of silicosis^15,17^. The combination of imaging-derived biomarkers with a persistent fibrotic mouse model and translational readouts such as the Tiffeneau index is a novel approach that allows assessment of disease progression and its stabilization over several weeks.

The choice of animal model to mimic human lung fibrotic disease and the way it is evaluated is essential for the translational relevance of mouse study results to man. At present, the most used and best-characterized animal model for lung fibrosis research and therapy evaluation is the more acute bleomycin model^6,26^. This model suffers from a high mortality rate and variability, is prone to survival bias and is claimed to be self-limiting in time^26^. The short timespan over which fibrosis develops and peaks does not allow truly therapeutic testing of interventions with antifibrotic potential, warranting gearing up to alternative, chronic models where fibrosis does not spontaneously resolve, such as irradiation-induced lung fibrosis^27,28^, transgenic mice and silicosis^29^. Silica instillation leads to fibrotic nodules that resemble those seen in patients exposed to environmental triggers such as dust and particulates^6^. When combined with longitudinal µCT, we here confirm that fibrosis is persistent in this model, offering a 10-week time frame allowing to test therapeutics^6,30^, two of the key aspects that the most often-used bleomycin model is lacking. Additionally, it is cheap, easy to perform, has a low inter-animal variability, zero mortality rate and a 100% success rate of fibrosis development.

Further in-depth characterization of the inflammatory and fibrotic response in this model revealed neutrophilic inflammation, peaking 1 week after silica instillation to slowly decrease to a neutrophilic inflammation of 20%, indicating a constant trigger of the immune system since particles are not easily cleared from the lungs by alveolar macrophages^31^. The neutrophilic inflammation is in concordance with several other studies^32,33^, although not as chronic as in our model, which might be due to differences in administration method, type and dose of silica and mouse strain used. Macrophages are the first to ingest silica particles, resulting in a cascade involving other inflammatory cells and several pro-inflammatory cytokines and chemokines^31,34^. We did not find an increased number of macrophages, though we did not evaluate macrophage activation status, nor macrophage sub-types. Studies have shown that both the M1 (pro-inflammatory) and M2 (anti-inflammatory/pro-fibrotic) subtypes play an important role in the silica-induced pathology^35^.

We found a variety of cytokines and chemokines in this silicosis model that have been implicated with pulmonary fibrosis. The increased concentration of TNF-α and IFN-γ suggests a pro-inflammatory response^15,36,37^. IL-13, mostly associated with wound-healing processes and considered pro-fibrotic, was increased during the entire disease process, indicating a type-2 response. Though Liu et al*.* suggest an induction of the Th2-response by crystalline silica particles^38^, we cannot conclude whether the increased IL-13 concentration is originating from Innate Lymphoid Cells type 2, Th2-cells or the epithelial cells since we did not formerly assess lymphocytes in lung tissue^39^. Type 3 cytokines IL-17A and IL-17F were elevated in our model as well as in other silica models^40^. All these readouts, together with the increased pro-fibrotic TGF-β cytokine, put forward an interplay between several innate and adaptive inflammatory (type 1, 2 and 3) and fibrotic processes over the entire time course of disease. We found an elevated SP-D concentration in serum, consistent with a similar finding in rats^41^. Alveolar type II and bronchiolar epithelial cells are responsible for the overproduction of SP-D after silica instillation and increased plasma SP-D levels have been suggested as an indication of lung injury and inflammation^31,42^. Together with the increased protein concentration in the BALF, this indicates epithelial damage due to the silica particles and a leaky epithelial barrier.

Several inflammatory and fibrotic indicators (protein concentrations in BAL, serum and OH-proline content) correlated with the non-aerated lung volume derived from the µCT scans. Moreover, lung function-derived tissue hysteresivity differences clearly indicate tissue inhomogeneity’s corresponding to inflammation and non-resolving fibrosis that also correlated with the µCT-derived non-aerated lung volume. We therefore propose the non-aerated lung volume as an alternative, non-invasive marker of disease burden consisting of intertwined fibrotic as well as inflammatory responses.

Where the non-aerated lung volume is a direct quantitative marker for the extent of pathology, the aerated lung volume is regarded a biomarker of lung function. Indeed, it correlated with volumetric readouts from endpoint lung function measurements such as inspiratory capacity. In this silicosis model, the aerated lung volume is enlarged during the plateau of the fibrotic phase, consistent with the absence of a lowered inspiratory capacity, FVC, FEV_0.1_ or increased elasticity; lung function readouts that would typically be affected in human restrictive disease or in the bleomycin model^8,10,43^.

The key to correct interpretation of lung function readouts lies in the consistently increased total lung volume retrieved from the µCT scans, reflecting enlargement of the lungs with silicosis (Fig. 2e). We interpret the enlargement of total lung volume, observed in this and other mouse models of lung diseases, as a compensation mechanism for the otherwise excessive loss of airspaces^11,21,44,45^. This phenomenon is absent in human patients, but in mouse models this enlargement of the lungs affects the interpretation of µCT-and lung function-derived readouts. Where lung function volumetric readouts may underestimate the presence of restrictive lung disease in this model, µCT offers readouts on the presence of inflammation and fibrosis and potential compensatory reaction thereto by enlargement of (aerated) lung volumes. Our findings here further emphasize the paramount importance of adding µCT examinations to the standard preclinical workflow to characterize the response of mouse models to lung insults.

Our imaging approach overcomes the limitations of standard endpoint assessments and lung function examinations, allows the individual monitoring of every animal throughout its entire disease process, thereby giving information with high statistical power on the extent of inflammatory and fibrotic processes. Including µCT-derived biomarkers in fibrosis research gives us important opportunities towards translation of preclinical study results given the availability of medical imaging data in routine patient care, and opens up avenues towards testing interventions therapeutically. This provides the necessary leap forward to move away from the currently most used prophylactic way of antifibrotic drug evaluation.

## Supplementary information

Supplementary Information 1.

Supplementary Information 2.
