# Supplementary material for: Longitudinal micro-computed tomography-derived biomarkers quantify non-resolving lung fibrosis in a silicosis mouse model
Source: Sci Rep. 2020 Sep 30;10:16181. doi: 10.1038/s41598-020-73056-6 (PMC7527558; doi:10.1038/s41598-020-73056-6)
Supplement: Supplementary file 1 — Supplementary Information 1. [file 41598_2020_73056_MOESM1_ESM.docx]

**Longitudinal micro-Computed Tomography-derived biomarkers quantify non-resolving fibrosis in a silicosis mouse model**

Kaat Dekoster^1^, Tatjana Decaesteker^2^, Nathalie Berghen^3,4^, Sofie Van den Broucke^5^, Anne-Charlotte Jonckheere^6^, Jens Wouters^1^, Anton Krouglov^1^, Rik Lories^3,4^, Ellen De Langhe^3,4^, Peter Hoet^5^, Erik Verbeken^7^, Jeroen Vanoirbeek^5#^, Greetje Vande Velde^1#*^

^1^ KU Leuven, Department of Imaging and Pathology, Biomedical MRI unit/MoSAIC, Leuven, Belgium

^2^ KU Leuven, Department of Chronic Diseases, Metabolism and Ageing, Lab of respiratory diseases, Belgium

^3^ KU Leuven, Department of Development and Regeneration, Skeletal Biology and Engineering Research Center, Leuven, Belgium

^4^ University Hospitals Leuven, Division of Rheumatology, Leuven, Belgium

^5^ KU Leuven, Department of Public Health and Primary Care, Centre for Environment and Health, Leuven, Belgium

^6^ KU Leuven, Department of Microbiology, Immunology and Transplantation, Allergy and clinical immunology research group, Leuven, Belgium

^7^ KU Leuven, Department of Imaging and Pathology, Translational Cell and Tissue Research Unit, Leuven, Belgium

# Last two authors share responsibility

* Corresponding author:

Prof. Greetje Vande Velde

KU Leuven

Department of Imaging and Pathology

Biomedical MRI unit/MoSAIC

Herestraat 49 mailbox 505

3000 Leuven, Belgium

Phone: +32 16 33 09 24

E-mail: greetje.vandevelde@kuleuven.be

**Supplementary information**

**Supplementary Figures**

**Figure S1: mouse weight after silica exposure.**

**Figure S2: percentage of neutrophils after silica exposure.**

At every endpoint, *ex vivo* readouts for inflammatory and fibrotic responses were obtained. Silica instillation changed the percentage of neutrophils. Data are presented as mean ± SD. Dashed line represents cross-sectional data. Two-way ANOVA with Sidak’s correction for multiple comparison was based on the following number of animals: w1 6 control and 6 silica mice; w5 6 control and 10 silica mice; w9 6 control and 8 silica mice; w15 6 control and 8 silica mice. ** p-value < 0.01; *** p-value < 0.001.

**Figure S3: additional pulmonary function readouts and their correlation with *in vivo* µCT-derived biomarkers.**

At every endpoint lung function was assessed by performing deep inflation, (a-c) forced oscillations and (g-i) negative pressure-driven forced expiration maneuvers. Data are presented as mean ± SD. Dashed line represents cross-sectional data. Two-way ANOVA with Sidak’s correction for multiple comparison was based on the following number of animals: w1 6 control and 6 silica mice; w5 6 control and 10 silica mice; w9 6 control and 8 silica mice; w15 6 control and 8 silica mice. * p-value < 0.05; ** p-value < 0.01; *** p-value < 0.001. Correlations between µCT-derived biomarkers and lung function measurements indicate the agreement between both methods (d) non- aerated lung volume and R, (e) mean lung density and G, (f) aerated lung volume and H, (j) mean lung density and PEF, (k) aerated lung volume and FVC, (l) mean lung density and FEV_0.1_. For every correlation the Pearson correlation coefficient is given and the p-value. Correlations only include silica-instilled animals.

**Figure S4: additional cytokines involved in inflammatory and fibrotic response measured in the BAL fluid.**

At every endpoint, different cytokine concentrations were determined. Silica instillation changed the amount of (a) IFN-γ, (b) IL-10, (c) IL-17A, (d) IL-17F, (e) IL-1β, (f) IL-6, (g) MCP-1, (h) TGF-β1, (i) TGF-β2 concentration in the BAL fluid. It did not significantly change the (j) TGF-β3 concentration in the BAL fluid. Data are presented as mean ± SD. Dashed line represents cross-sectional data. Two-way ANOVA with Sidak’s correction for multiple comparison was based on the following number of animals: w1 6 control and 6 silica mice; w5 6 control and 10 silica mice; w9 6 control and 8 silica mice; w15 6 control and 8 silica mice. For TGF-β1-2-3 analysis, the following number of animals was used: w1 6 control and 8 silica mice; w5 6 control and 9 silica mice; w9 6 control and 8 silica mice; w15 6 control and 8 silica mice. * p-value < 0.05; ** p-value < 0.01; *** p-value < 0.001.
