# Supplementary material for: Longitudinal micro-computed tomography-derived biomarkers quantify non-resolving lung fibrosis in a silicosis mouse model
Source: Sci Rep. 2020 Sep 30;10:16181. doi: 10.1038/s41598-020-73056-6 (PMC7527558; doi:10.1038/s41598-020-73056-6)
Supplement: Supplementary file 2 — Supplementary Information 2. [file 41598_2020_73056_MOESM2_ESM.docx]

**Longitudinal micro-Computed Tomography-derived biomarkers quantify non-resolving fibrosis in a silicosis mouse model**

Kaat Dekoster^1^, Tatjana Decaesteker^2^, Nathalie Berghen^3,4^, Sofie Van den Broucke^5^, Anne-Charlotte Jonckheere^6^, Jens Wouters^1^, Anton Krouglov^1^, Rik Lories^3,4^, Ellen De Langhe^3,4^, Peter Hoet^5^, Erik Verbeken^7^, Jeroen Vanoirbeek^5#^, Greetje Vande Velde^1#*^

^1^ KU Leuven, Department of Imaging and Pathology, Biomedical MRI unit/MoSAIC, Leuven, Belgium

^2^ KU Leuven, Department of Chronic Diseases, Metabolism and Ageing, Lab of respiratory diseases, Belgium

^3^ KU Leuven, Department of Development and Regeneration, Skeletal Biology and Engineering Research Center, Leuven, Belgium

^4^ University Hospitals Leuven, Division of Rheumatology, Leuven, Belgium

^5^ KU Leuven, Department of Public Health and Primary Care, Centre for Environment and Health, Leuven, Belgium

^6^ KU Leuven, Department of Microbiology, Immunology and Transplantation, Allergy and clinical immunology research group, Leuven, Belgium

^7^ KU Leuven, Department of Imaging and Pathology, Translational Cell and Tissue Research Unit, Leuven, Belgium

# Last two authors share responsibility

* Corresponding author:

Prof. Greetje Vande Velde

KU Leuven

Department of Imaging and Pathology

Biomedical MRI unit/MoSAIC

Herestraat 49 mailbox 505

3000 Leuven, Belgium

Phone: +32 16 33 09 24

E-mail: greetje.vandevelde@kuleuven.be

**Supplementary information**


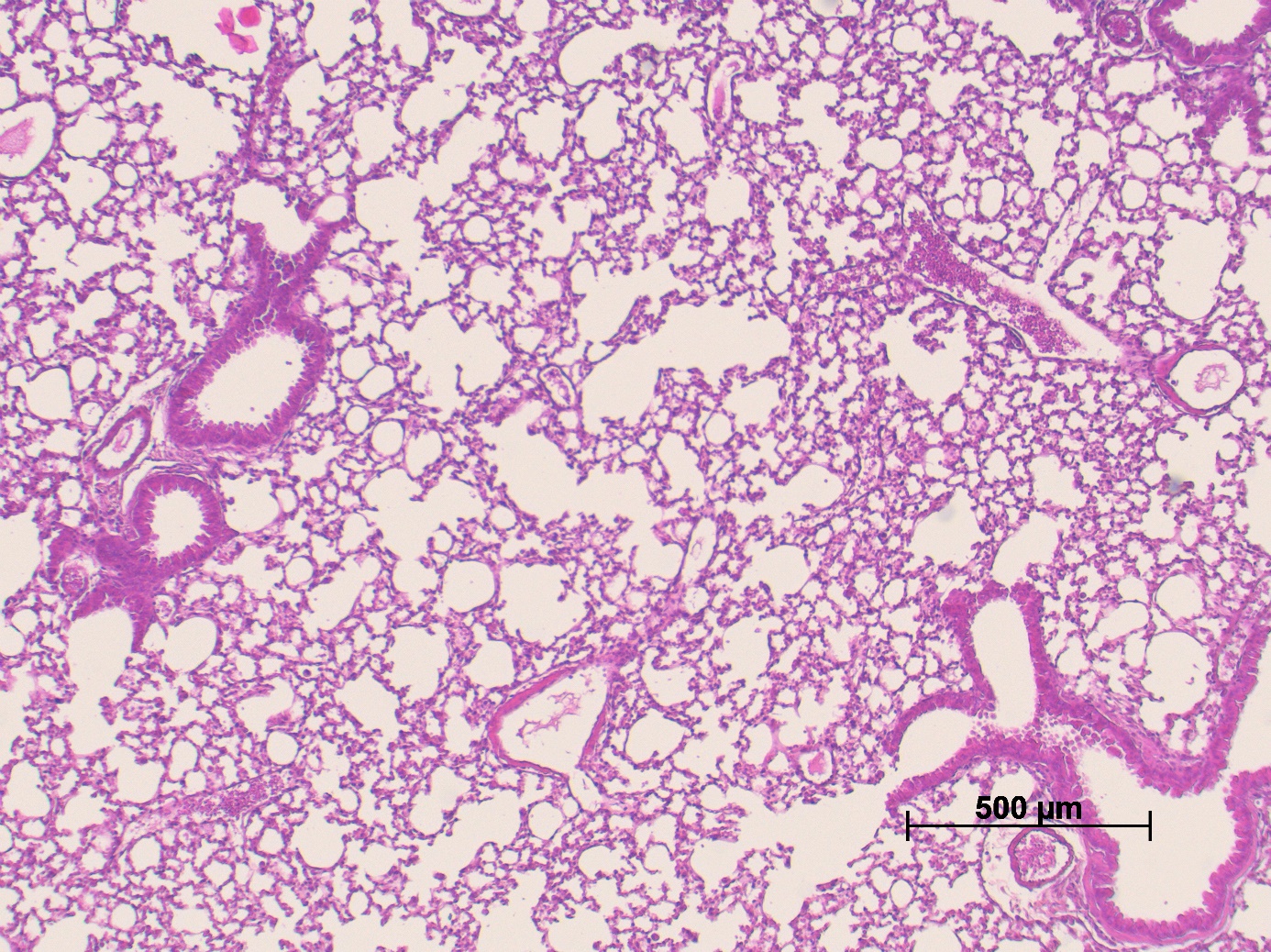


**Figure S5A: histopathological analysis of control-instilled animal week 1.**

Representative H&E stained images are shown for control animal (magnification 50x).


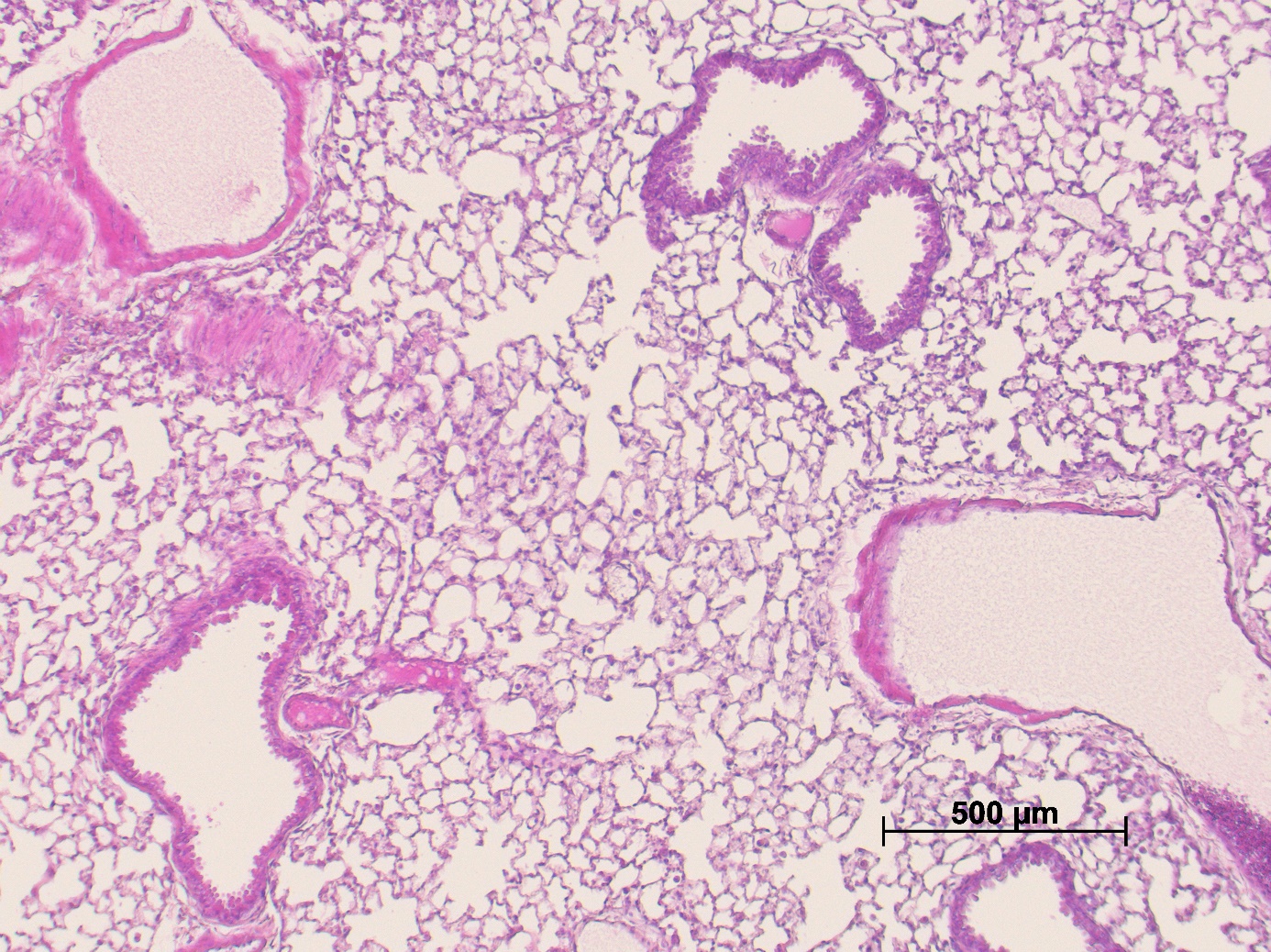


**Figure S5B: histopathological analysis of control-instilled animal week 5.**

Representative H&E stained images are shown for control animal (magnification 50x).


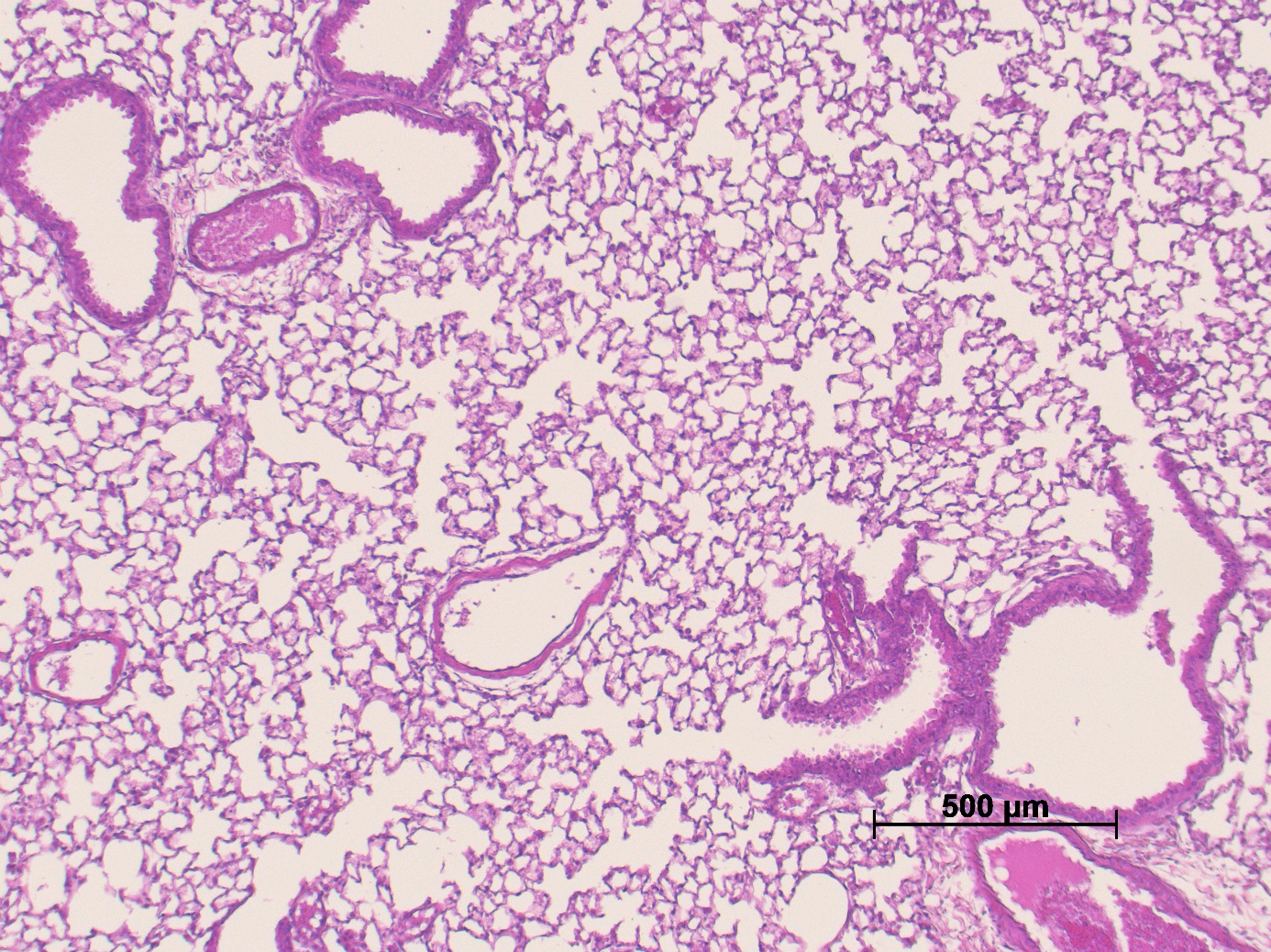


**Figure S5C: histopathological analysis of control-instilled animal week 9.**

Representative H&E stained images are shown for control animal (magnification 50x).


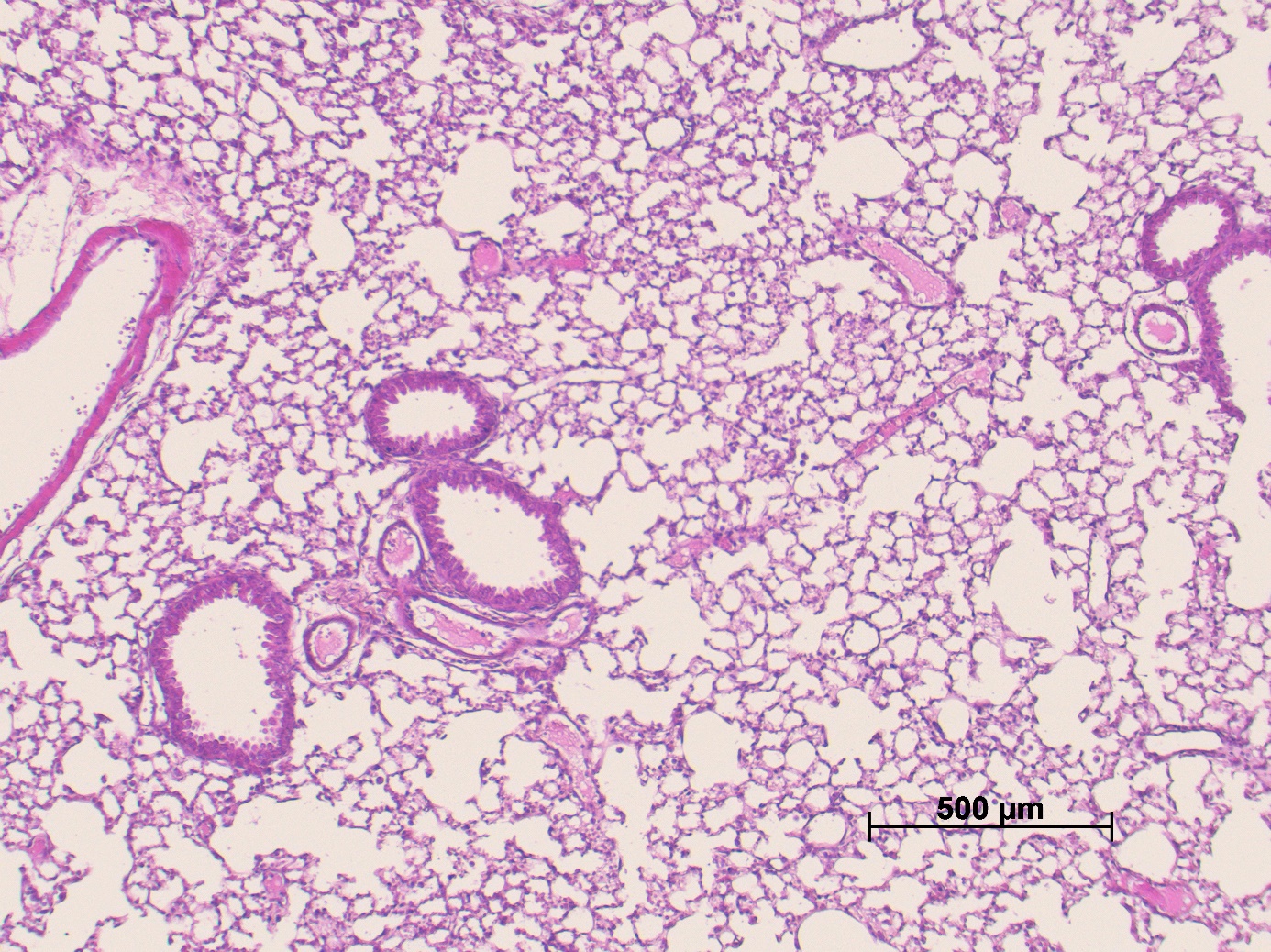


**Figure S5D: histopathological analysis of control-instilled animal week 15.**

Representative H&E stained images are shown for control animal (magnification 50x).


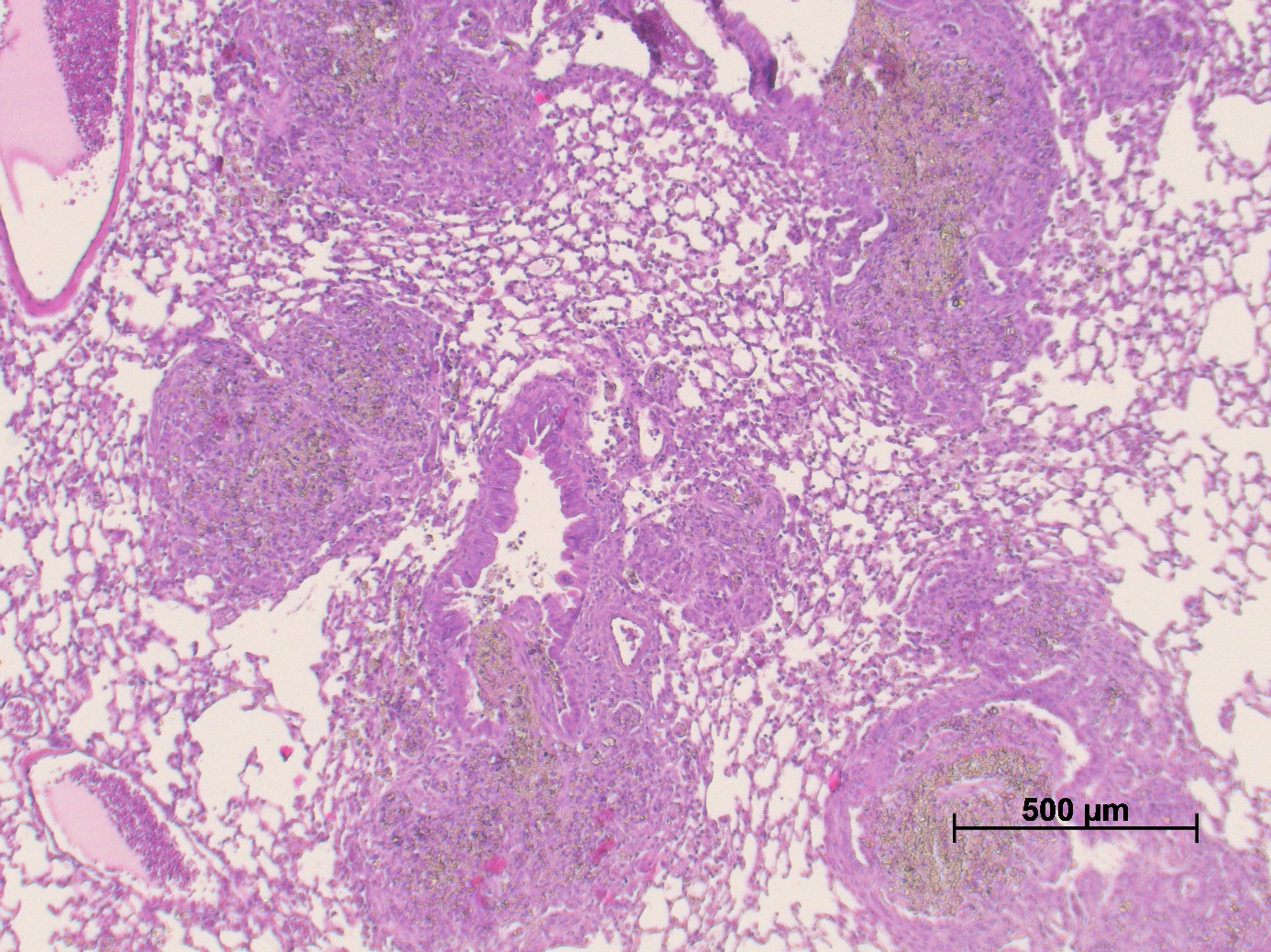


**Figure S5E: histopathological analysis of silica-instilled animal week 1.**

Representative H&E stained images are shown for silica-instilled animal (magnification 50x).


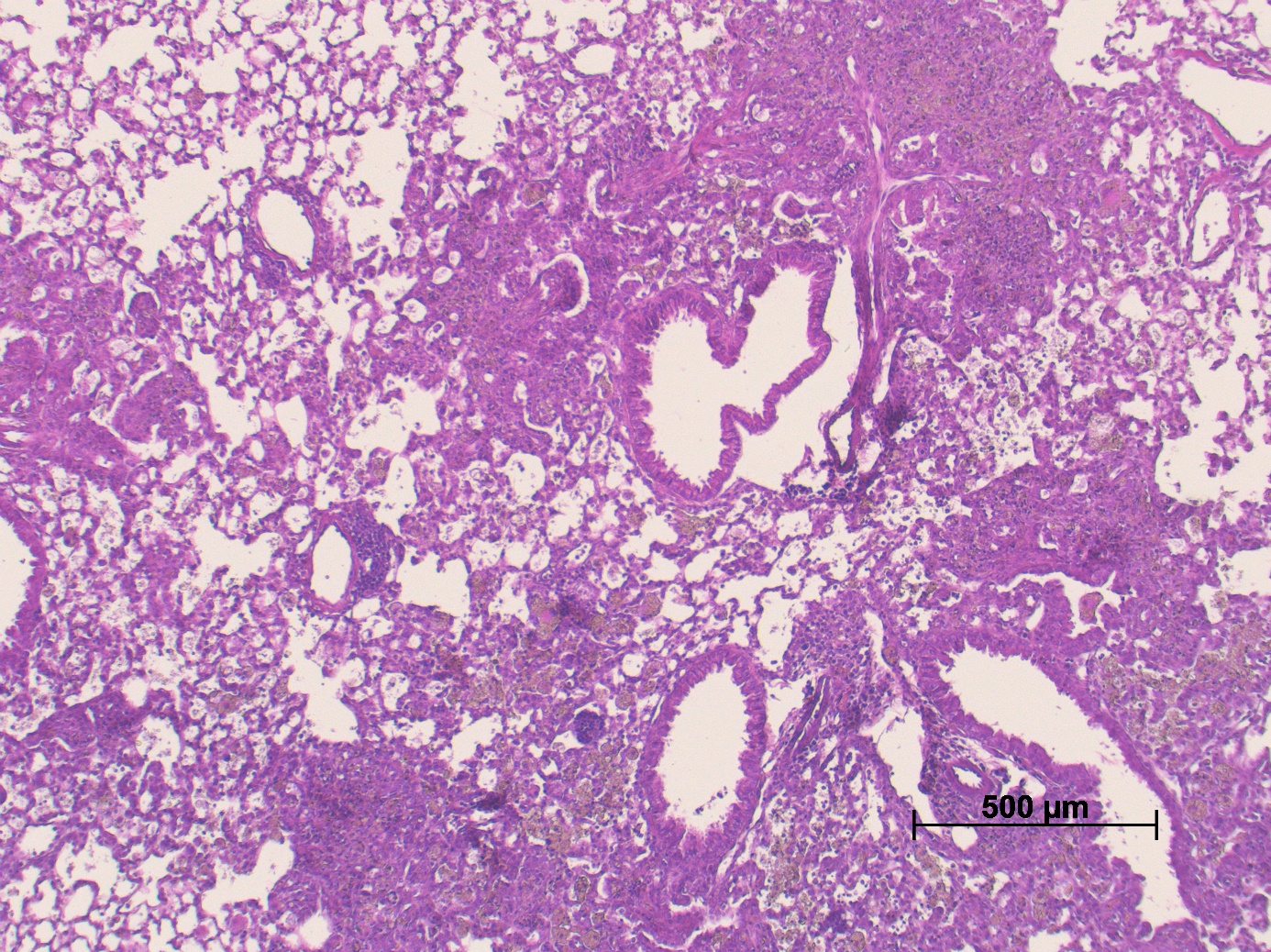


**Figure S5F: histopathological analysis of silica-instilled animal week 5.**

Representative H&E stained images are shown for silica-instilled animal (magnification 50x).


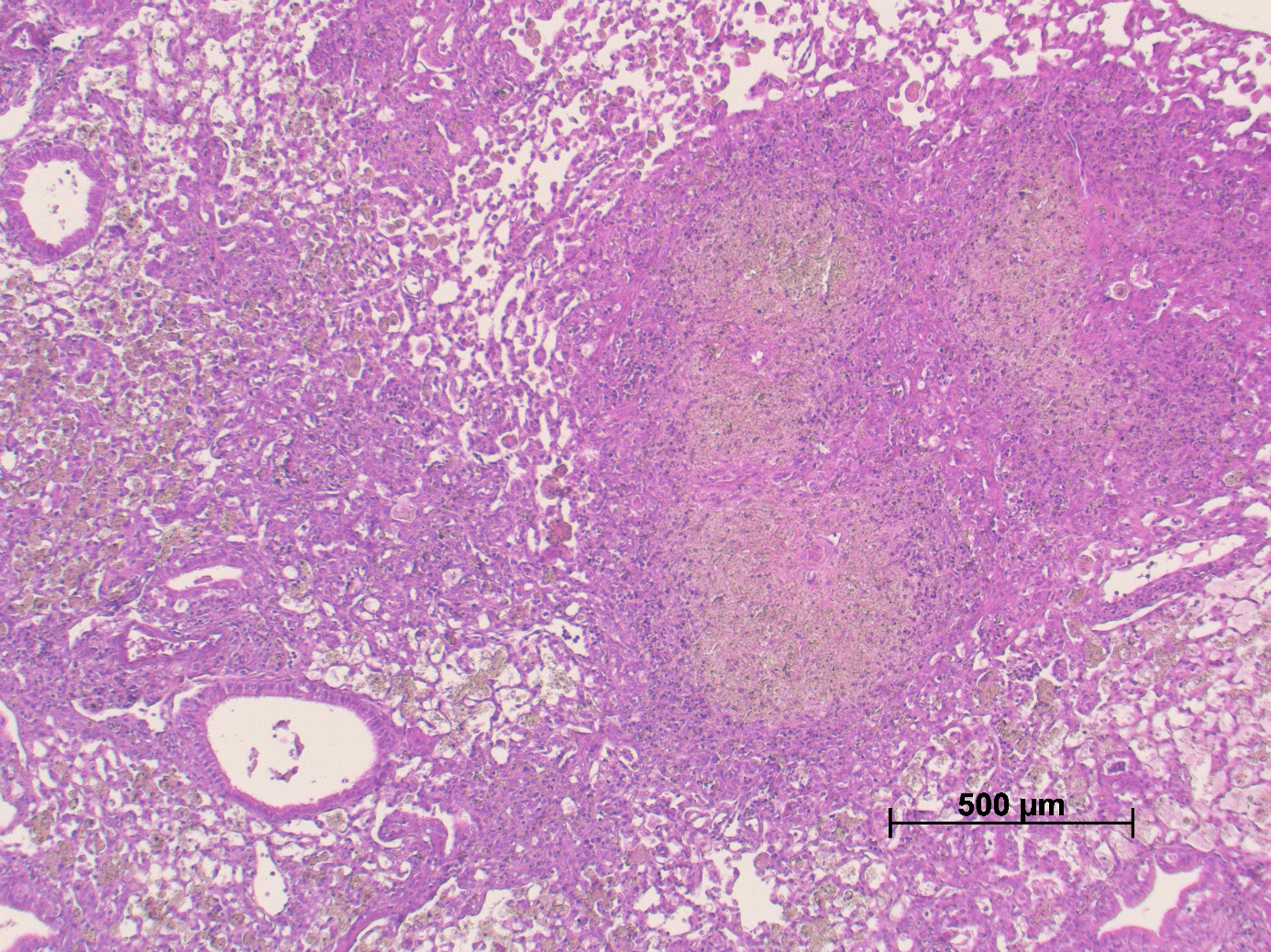


**Figure S5G: histopathological analysis of silica-instilled animal week 9.**

Representative H&E stained images are shown for silica-instilled animal (magnification 50x).


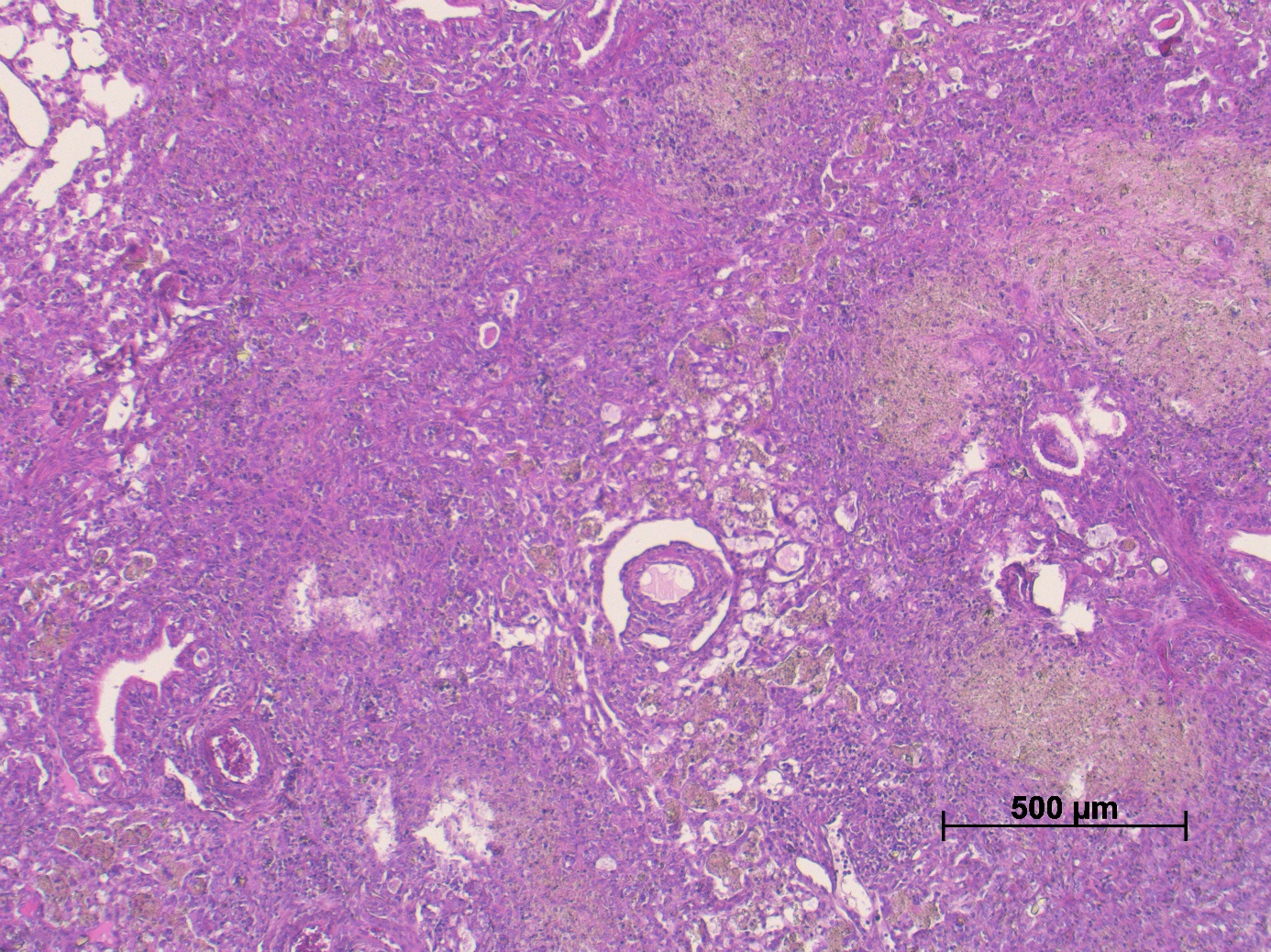


**Figure S5H: histopathological analysis of silica-instilled animal week 15.**

Representative H&E stained images are shown for silica-instilled animal (magnification 50x).
